# Supplementary material for: Stress and burnout amongst mental health professionals in Singapore during Covid-19 endemicity
Source: PLoS One. 2024 Jan 11;19(1):e0296798. doi: 10.1371/journal.pone.0296798 (PMC10783734; doi:10.1371/journal.pone.0296798)
Supplement: Attachment — Submitted filename: Responses to reviewers.docx [file pone.0296798.s002.docx]

Dear Editor and reviewers,

Thank you for your feedback and suggestions. Please find below the responses to each point raised by the editor/ reviewers.

**Responses to editor**

Please correct step by step all comments from the reviewers, as well you can mention how political decisions that are made affect burnout.

Response: Thank you for highlighting the importance of health politics. We have added more suggestions on how health policy can improve the well-being of MHP in p19 (Discussion section).

**Responses to reviewer#1**

Reviewer #1: Thank you for allowing me to review this interesting paper on stress and burnout amongst mental health professionals in Singapore during COVID-19 endemicity. As COVID-19 is part of our everyday lives currently, and as mental health issues gain prominence, making sure that mental health professionals who provide care to patients that need this help is very important. This paper has the potential to inform clinical practice.

I have a few minor suggestions for the authors to consider:
1. In the statistical section, state what p value as considered significant.

Response: Thank you for the feedback and suggestions. We have added in “P-value <0.05 was used to determine whether the ANOVA/ post-hoc tests were statistically significant” in p7 (Data analysis section).

2. It would be helpful to provide some context on the clinical workload of the centre, including how this changed during the different time periods, even if discussed in a qualitative fashion that would be helpful.

Response: Thank you for the suggestion. We have added in some context on the clinical workload of MHP and how it changed during the different time periods in p13-14 (Discussion section).

3. Discuss the potential impact of the limitations on the estimates seen in the results.

Response: We are unsure what estimates you are referring to here. We have indicated in the limitation section that due to low response rate, finding cannot be generalised to all MPH working in Singapore). We have also added that use of different sample cohort limits interpretation of causality and a longitudinal follow-up study is recommended. These can be found in p 19 (Limitation section).

**Responses to reviewer#2**

Reviewer #2: please refer to my reviewers comments that i have attached above.
I think this it is an important topic. The study aimed to highlight the high prevalence of burn out and stress in the mental health professionals. Nonetheless the low response rate of the survey limits the strength of the study to draw meaningful conclusions. The comparative data was drawn from previous publications without elaborations of consistency of methodology or study population
Authors should also consider drawing references from previous multi-institutional, longitudinal study that were conducted in similar healthcare setting.

Response to item 1: Thank you for your feedback and suggestions. The first study in May 2020 was carried out 2 months after strict lockdown measures were implemented in April 2020 in Singapore. The aim was to collect baseline burnout data among healthcare workers. The second study in September 2021 was carried out after about 80% of the population have been vaccinated against COVID-19 by August 2021. Despite the high vaccination rate in Singapore, the healthcare system was still strained by a high volume of COVID-19 cases and it was unclear whether there was any change in burnout rate among healthcare workers. The aim of the September 2021 study was to compare the burnout rate to the baseline rate. The third study in June 2022 was carried out 6 months after Singapore has transited into a state of COVID-19 endemicity. The aim was to assess whether burnout among healthcare workers has lessened to an acceptable level during COVID-19 endemicity. We have added a summarized timeline of the major covid related events in p.3-4 (Introduction section). This will help provide context to the timing of the 3 studies.

Response to item 2: The decision to implement the study was based on major COVID-19 related events as explained in point 1. We did not time our studies based on when the COVID-19 waves spikes as these spikes are hard to predict. We have added how some COVID-19 related events might have an impact on MHP burnout rate in p.13-14 (Discussion section)

Response to item 3: The possible factors that may aggravate or mitigate stress and burnout were drawn from a study conducted in the same psychiatry hospital (IMH) in 2014 by the same author. Though only the quantitative findings were published then, the qualitative findings were analyzed internally to provide a better understanding of causes of burnout among mental health professionals. These findings were contributed by mental health professionals working in the same workplace (with similar work conditions and work culture) and provide valuable and relevant insights into possible factors that may aggravate or mitigate stress and burnout for mental health professionals working in IMH. Thus they were used to formulate the survey question in this study.

In the discussion section, we have referenced some studies from Asian region when discussing factors that impact stress and burnout. Please refer to page 16-17. Thank you for bringing our attention to Teo el al study. We have added a discussion point in reference to this study in p16 (Discussion section).

Response to item 4: Yes, we agree that the low response rate and the use of different sample cohorts to trend the burnout rate is a limitation to the study. We have added these limitations in p 19 (Limitation section).
