# Supplementary material for: Stress and burnout amongst mental health professionals in Singapore during Covid-19 endemicity
Source: PLoS One. 2024 Jan 11;19(1):e0296798. doi: 10.1371/journal.pone.0296798 (PMC10783734; doi:10.1371/journal.pone.0296798)
Supplement: Attachment — Submitted filename: Response to reviewers.docx [file pone.0296798.s003.docx]

Dear Editor and reviewers,

Thank you for your feedback and suggestions. Please find below the responses to each point raised by the reviewers.

**Responses to reviewer#4**

Overall, there are grammatically some incorrect parts (Line 369:exitant,line 91:this 2 months, line 266: exitant, line108:April2022, line 112 :June2022)->grammatical correction required

The above listed grammatical errors have been corrected.

Methodology
-The author should show a sampling method used (how participants have been chosen from population) and the period of data collection

All staffs working at IMH were send the email with the survey link and the period of data collection was one month. These are now added in the methodology (data collection) section.

-it would be good to add Ethical part that would gather clear information on ethic (from line 106-108 and Line 115-117). Some informations of ethic appear in the study methods and data collection.

Line 115-117 have been moved to the ethical section (starting with line 106) as suggested.

-I would like to know if the data extracted from burnout scores from two early multi-centre studies were only about burnout, as there is no information on stress from the two studies?

Yes, the earlier 2 studies did not collect stress (PSS) data.

-It would good to state the statistical method used on each specific objective, as there is information on the statistical method used to assess factors contributing to stress and burnout and areas of improvement.

We have added in line103 that the method used to assess factors contributing to stress and burnout is ANOVA in line 106.

-There is a need of explanation in methodology part on how analysis has been made on each items(stress and burnout) as it seems the three time comparison has been made on burnout only while for stress the information is given on the survey conducted by the author in this study(year 2022)

As the earlier 2 studies did not collect stress (PSS) data, only burnout (OLBI) data is available for comparison across 3 timepoint. We have added this in line 125-127.

Discussion

In reducing the discussion part Somme parts should be removed. For example the information given in lines 350-353 and lines 364-366 should be removed or moved at the last paragraph.

Line 350-353 have been removed as suggested. However, we have kept line 364-366 (line 371-373 in the revised manuscript with track change) as it is useful information to know that an external help service is now available and that further investigation into its effectiveness is needed in the future. This information fits better in its current place than in the last paragraph.
